# Transcatheter closure of ventricular septal rupture with prolonged support of intra-aortic balloon pump after primary PCI: a case report

**DOI:** 10.1186/s12872-021-02392-w

**Published:** 2021-12-18

**Authors:** Chuan Yang, Yong Sun, Deling Zou, Zhaoqing Sun, Xinzhong Zhang, Guangsheng Su, Jing Qi, Wenyue Pang

**Affiliations:** 1 Department of Cardiology, Shengjing Hospital of China Medical University, 36 Sanhao Street, Shenyang, 110004 China; 2 Department of Cardiology, The Second Affiliated Hospital of Harbin Medical University, Harbin, 150086 China; 3 The Key Laboratory of Myocardial Ischemia, Harbin Medical University, Ministry of Education, Harbin, 150086 China

**Keywords:** Acute myocardial infarction, Complication, Ventricular assist device, Intervention, Case report

## Abstract

**Background:**

Ventricular septal rupture (VSR) is a rare but severe complication of acute myocardial infarction (AMI). For such cases, surgical repair is recommended by major guidelines, but not always possible for such cases.

**Case presentation:**

A 72-year-old man presented to the emergency room. ECG showed the ST-segment was elevated by 2–3 mm in lead II, III, and aVF, with Q-waves. Coronary angiography (CAG) showed multi-vessel disease with a total occlusion of the right coronary artery (RCA) and severe stenosis of the left anterior descending artery (LAD). A diagnosis of acute inferior myocardial infarction was made. VSR occurred immediately after percutaneous coronary intervention (a 2.5 × 20 mm drug-eluting stent implanted in RCA), and the patient developed cardiogenic shock. An intra-aortic balloon pump (IABP) was used to stabilize the hemodynamics. Transthoracic echocardiography (TTE) revealed an 11.4-mm left-to-right shunt in the interventricular septum. An attempt was made to reduce the IABP augmentation ratio for weaning on day 12 but failed. Transcatheter closure was conducted using a 24-mm double-umbrella occluder on day 28. The patient was weaned from IABP on day 31 and underwent secondary PCI for LAD lesions on day 35. The patient was discharged on day 41. Upon the last follow-up 6 years later, CAG and TTE revealed no in-stent restenosis, no left-to-right shunt, and 51% left ventricular ejection fraction.

**Conclusions:**

Prolonged implementation of IABP can be a viable option to allow deferred closure of VSR in AMI patients, and transcatheter closure may be considered as a second choice for the selected senior and vulnerable patients, but the risk is still high.

## Background

Ventricular septal rupture (VSR) is a rare but severe complication of acute myocardial infarction (AMI). The 30-day mortality rate of VSR is approximately 80%. The incidence of VSR has substantially decreased since the widespread implementation of early reperfusion strategies, including primary percutaneous coronary intervention (PCI) and thrombolytic treatment, with a current estimate between 0.17% and 0.34% [1, 2]. VSR occurs most often within the first 24 h after AMI [3], and the incidence of VSR is lower in patients who receive primary PCI compared with those who undergo delayed or elective PCI after a recent AMI [4].

Major guidelines recommend surgical repair in patients who develop VSR after AMI [5]. Transcatheter closure is a second choice after surgery, and it is implemented for congenital heart defect as several occluding devices have been developed over the past 2 decades [6]. Some researchers have demonstrated that transcatheter closure after VSR is possible and effective in the deferred period of the rupture [7].

Intra-aortic balloon pump (IABP) is commonly used in patients with cardiogenic shock to temporarily stabilized hemodynamic disturbances [8]. It can reduce the afterload of the left ventricle to decrease myocardial oxygen demand and increase the peak diastolic pressure. As a result, it can enhance coronary, cerebral, and renal perfusion, then subsequently raise cardiac output [9]. A previous study showed that IABP could reduce the left-to-right shunt in patients with VSR caused by AMI [10].

Here, we report a case of IABP use for prolonged time before percutaneous closure of VSR after primary PCI. Surgical repair was once considered but abandoned because of a higher risk of anesthesia, extracorporeal circulation support, and bleeding when dual anti-platelet therapy (DAPT) was adopted. Some previous reports showed cases of postinfarct VSR receiving immediate surgical or transcatheter closure operation had bad outcomes, and other reports presented cases received successful closure operation and revascularization after a deferred period [7, 11–13]. Some observational studies show early intervention is a risk factor for operation-related mortality [12, 14, 15]. So, the current issue is that some postinfarct VSD patients need a period of recovery and healing prior to operational intervention, but such patients may not wait for such a long-deferred time due to the poor hemodynamic status and heart function. The IABP may be the solution to fill the gap period to support those patients who need the waiting time for the deferred operation. A previous published study has reported implementation of IABP and then percutaneous closure of VSR [16]. In our case, the occurrence of VSR happened after primary PCI, and an immediate decision of IABP implementation was made in the emergent situation, then the IABP was prolonged used for 4 weeks prior to transcatheter closure of the VSR without any complication. The patient provided written informed consent for publication of this report. We present the following case in accordance with the CARE reporting checklist.

## Case presentation

**Fig. 1:**
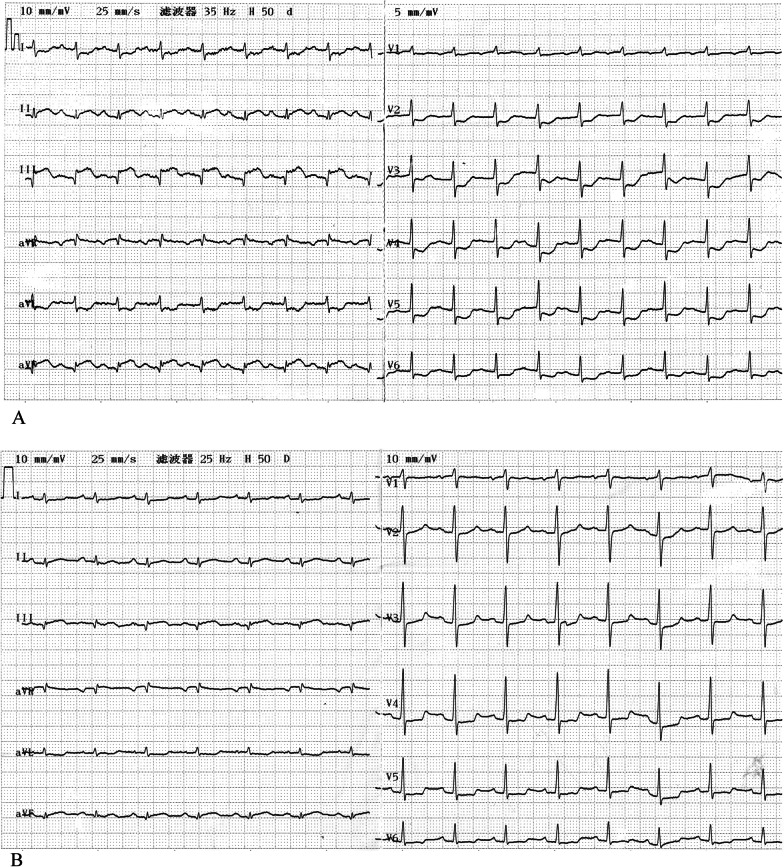
**A** Upon admission: ECG showed sinus rhythm with heart rate of 113 bpm and ST-segment elevation in leads II, III, aVF with Q-waves. **B** ECG after primary PCI showed attenuation of ST-segment elevation and the depths of the Q-waves

A 72-year-old man presented to emergency room with 8-h fatigue and 4-h mild exertional dyspnea, palpitation, and blurred vision. He was a nonsmoker and denied previous history of cardiovascular disease. Physical examination showed sinus tachycardia (117 bpm) and normal blood pressure (120/70 mmHg). The position and range of apical impulse were normal. There were no heart murmurs, no crackles or wheezes on chest auscultation. In ECG, the ST-segment was elevated by 2–3 mm in leads II, III, and aVF, with Q-waves (Fig. 1A). Cardiac troponin T was 3.33 ng/mL (normal range 0–0.04). A diagnosis of acute inferior myocardial infarction was established.

**Fig. 2:**
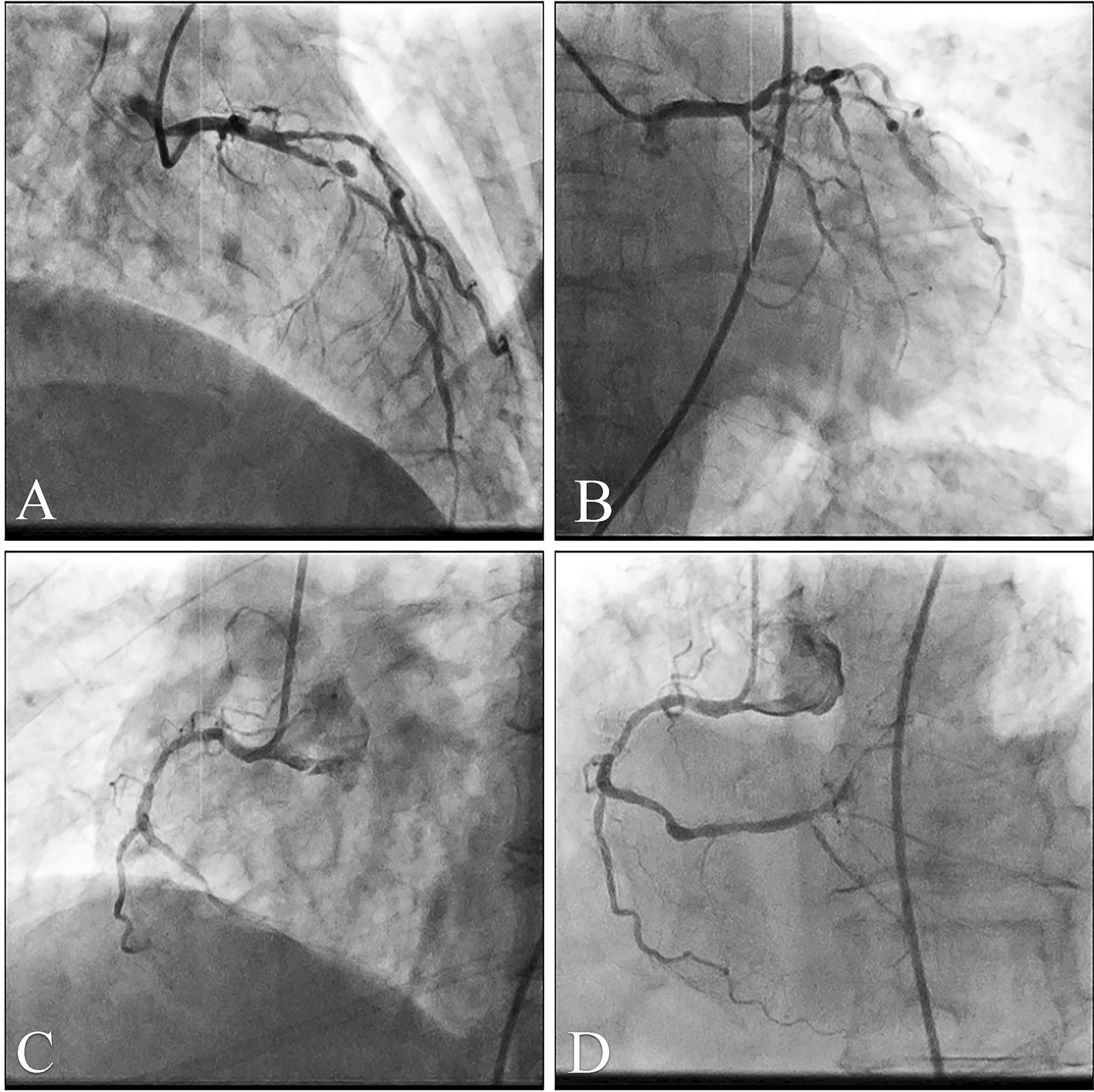
Coronary angiography. **A** 90% severe stenosis and aneurysmal dilatation of LAD; cranial view. **B** Diffuse stenosis (50–60%) of LCX; caudal view. **C** Total occlusion of the distal portion of an RCA. **D** RCA after primary PCI

Dual anti-platelet therapy (loading doses: aspirin 300 mg and clopidogrel 300 mg) was initiated to prepare for primary PCI. Coronary angiography (CAG) showed multi-vessel lesions, including a total occlusion of the distal portion of a dominant right coronary artery (RCA), 90% stenosis of the proximal portion of the left anterior descending artery (LAD), and diffuse stenosis (50–60%) of the left circumflex artery (LCX) (Fig. 2A–C). The culprit lesion was in the distal portion of the RCA. The patient received a loading dose of glycoprotein IIb/IIIa inhibitor (tirofiban) after the angiography. Percutaneous balloon angioplasty was then conducted; one BuMA^TM^ 2.5 × 20 mm sirolimus-eluting stent was placed to restore blood flow in RCA (TIMI grade 3) (Fig. 2D). ST-segment elevation and depths of the Q-waves were attenuated after the primary PCI (Fig. 1B). Secondary PCI was planned for LAD lesions 5 days later.

Immediately prior to transfer to the cardiac intensive care unit (CCU), the patient developed severe dyspnea. Heart rate (HR) was 120 bpm, and blood pressure (BP) decreased to 90/60 mmHg. Auscultation revealed a loud harsh holosystolic murmur along the left sternal border and crackles throughout both lungs. NYHA functional class of heart failure was IV. A mechanical complication was suspected, but not investigated immediately. We immediately placed an IABP (1:1 augmentation ratio) from femoral access, and the patient received 3-mg morphine, 20-mg furosemide, and 0.1-mg recombinant human brain natriuretic peptide (rhBNP). Once the IABP was inserted, symptoms became alleviated. The BP stabilized at 110/70 mmHg and the augmentation pressure was 120 mmHg. Then the patient was transferred to CCU safely.

**Fig. 3:**
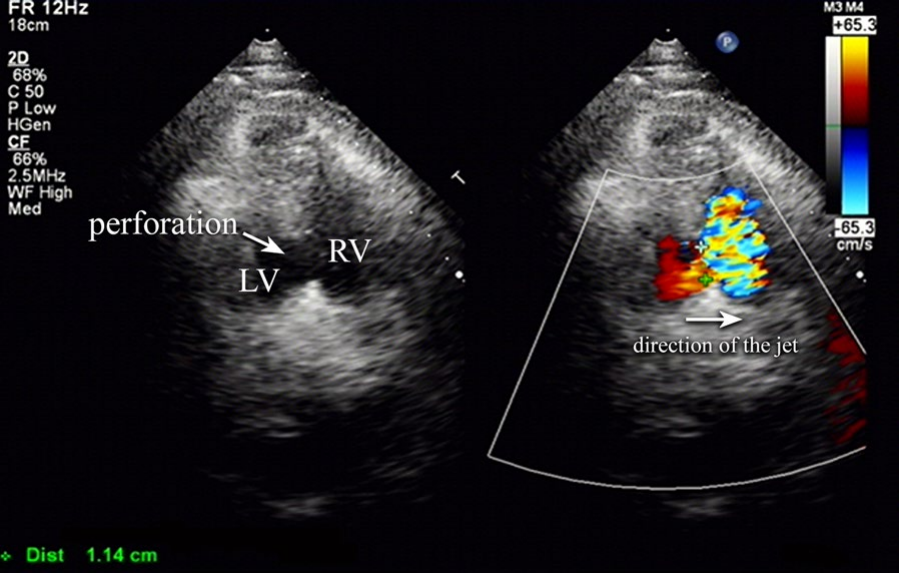
Transthoracic echocardiography: ventricular short-axis view with (right) and without (left) color-Doppler. A left-to-right shunt was detected in the level of ventricular level. The left ventricle is on the left-hand side (LV), so in the right ventricle (RV), a colorful signal of blood flow (downstream of the jet) can be displayed. The arrow on the left shows the location of the VSR, which is in dark color, and the arrow on the right denotes the direction of the jet. The Qp/Qs was 2.905 after the calculation

Transthoracic echocardiography (TTE) was performed the next day and revealed a left-to-right shunt in the posterior portion of the interventricular septum with a size of 11.4 mm (Fig. 3). The left ventricle end-diastolic diameter (LVED) was 50 mm, and the diameter of the right ventricle was 20 mm. We also detected regional wall-motion akinesia of the left ventricle in the inferior and posterior sections. Left ventricular ejection fraction (LVEF) was 59%. The estimated pulmonary arterial pressure and right ventricular systolic pressure were 48 mmHg. A calculated Qp/Qs was 2.905. There was no regurgitation of the mitral valve or pericardial effusion. An updated diagnosis of ventricular septal rupture was made. We decided to continue IABP to support cardiac function. Medications included aspirin (100 mg orally once a day), clopidogrel (75 mg orally once a day), atorvastatin (20 mg orally once a day), furosemide, spironolactone, and nitrates, as well as an intravenous infusion of rhBNP. The patient received a subcutaneous injection of 40-mg enoxaparin every 12 h to prevent deep vein thrombosis. Omeprazole (40 mg per day) was used to prevent gastrointestinal mucosal injury.

An attempt to wean IABP was made on day 12 on the ground of stable hemodynamics and disappearance of all symptoms. Five minutes after reducing the augmentation ratio from 1:1 to 1:2, the patient felt dyspnea, and the BP decreased from 116/68 mmHg to 71/50 mmHg. The augmentation ratio was increased back to 1:1. Dyspnea gradually dissipated, and the BP normalized. We realized the shunt was in a large amount according to the Qp/Qs. IABP can decrease the shunt, so we decided to implement a prolonged use of IABP, which continued at 1:1 ratio for another 16 days. During the use of IABP, we enhanced the medical and nursing concern including checking the circulatory status of the lower limb of the puncture with Doppler ultrasound every day, limiting its movement to avoid dislocation of the balloon catheter and bleeding, and continuous administration of anticoagulation by subcutaneous injection of enoxaparin to prevent deep vein thrombosis. The aspirin, clopidogrel, atorvastatin, spironolactone, and rhBNP were administered during the month when IABP was used. An antibiotic (cefamandole 2000 mg intravenously every 12 h) was given for prophylaxis for bacteremia during invasive IABP insertion from day 21 to 27. Psychological therapy was adopted to enable the patient to cooperate on prolonged use of IABP.

Surgical repair was offered to the patient on day 27 since the friable tissue in the ischemic myocardium should be sufficiently mature at this time to allow repair. However, the surgeons considered that it was too risky to perform the surgical repair due to the limitation of technique at that time. Firstly, the VSR located at the posterior muscular part of the ventricular septum was lower and deeper than a perimembranous ventricular septal defect approached through a right atriotomy and the tricuspid valve, so the surgical repair was more difficult and beyond the techniques of the surgeons, when they only had experience in the treatment of perimembranous ventricular septal defects at that time. Secondly, the EuroSCORE [17] of this case they calculated was 16, and the predicted mortality was 59.07%, which was too high for them to operate safely. Meanwhile, we also consulted the anesthesiologist to evaluate the patients for general anesthetization, and the anesthesiologists deemed there would probably be high risk in the process of a general anesthesia for the open surgery and especially under extracorporeal circulation support. Surgery was also declined by the patient and his family due to the high risk of open surgery and under general anesthesia and extracorporeal circulation support. Instead, the patient opted to receive percutaneous closure of the VSR. So we decided to choose percutaneous VSR closure for this specific high-risk senior patient.

**Fig. 4:**
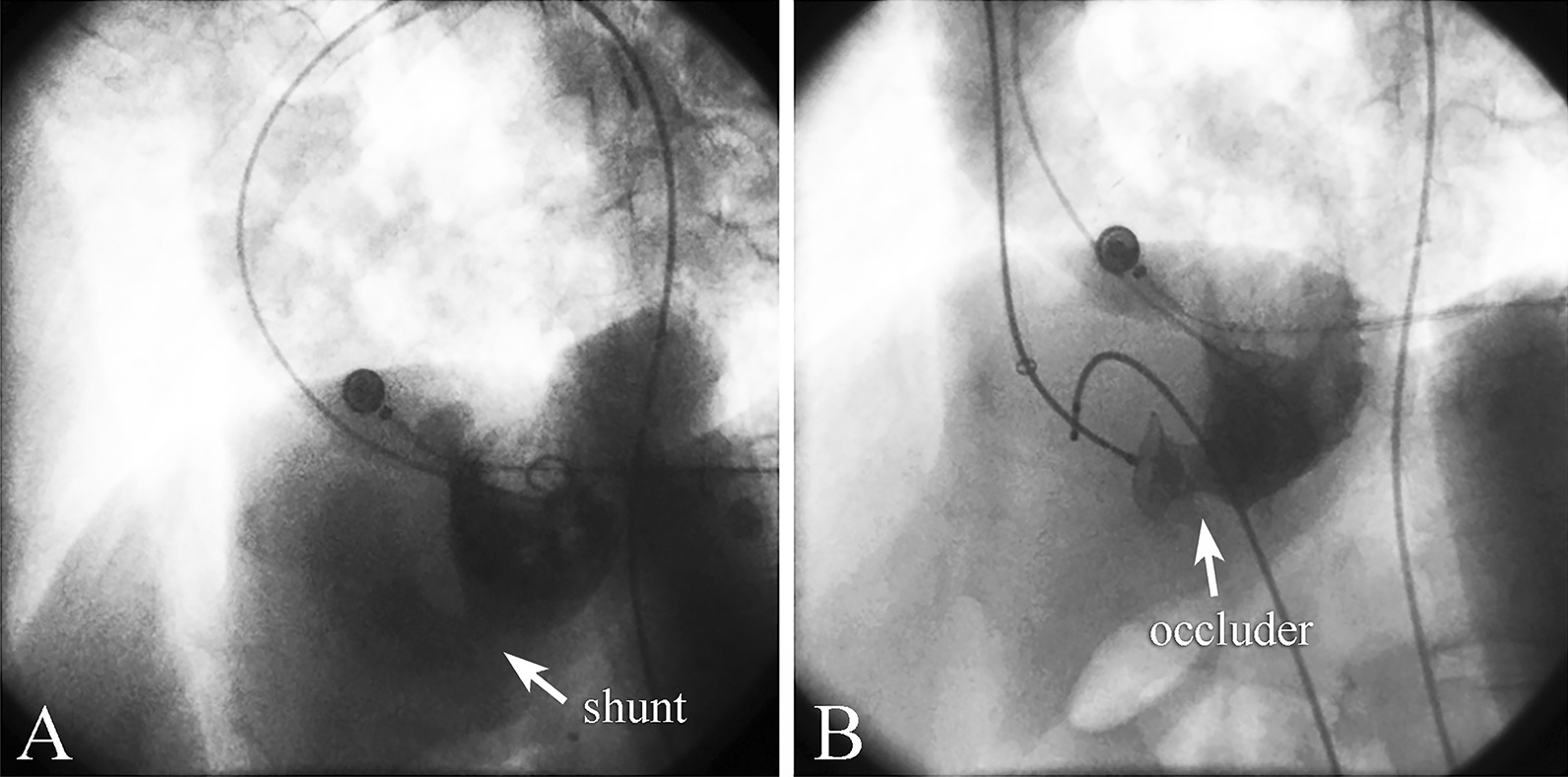
**A** Left ventriculography showed the left-to-right shunt. The arrow denotes the position of the shunt. **B** Immediately after transcatheter closure with a 24-mm occluder

On day 28 (4 weeks after VSR), the patient didn’t feel dyspnea or any other discomfort with stable vital signs (HR 90 bpm, BP 98/67 mmHg, augmentation pressure 112 mmHg). We believed the waiting period for the infarcted myocardium to recover was enough according to the documented experiences [18, 19]. Left ventriculography confirmed an 11-mm left-to-right shunt (Fig. 4A). Transcatheter closure was conducted via the femoral artery and subclavian vein. Upon establishment of the transseptal wire loop as a rail, the patient developed ventricular fibrillation, and he lost consciousness followed by. A 200-J electrical shock was delivered immediately and restored sinus rhythm. Then the patient regained consciousness. After the wire loop through the rupture was established, the HR suddenly decreased to 30 bpm. A temporary pacemaker was placed, and we proceeded to implant a 24-mm double-umbrella AGA AMPLATZER^TM^ occluder to close the VSR (Fig. 4B). The vital signs were stable (HR 78 bpm, BP 110/72 mmHg). Echocardiography after the procedure revealed no residual shunt, and no interference of valve functions by the occluder. The estimated Qp/Qs was 1.086. The temporary pacemaker was removed after the closure operation.

**Fig. 5:**
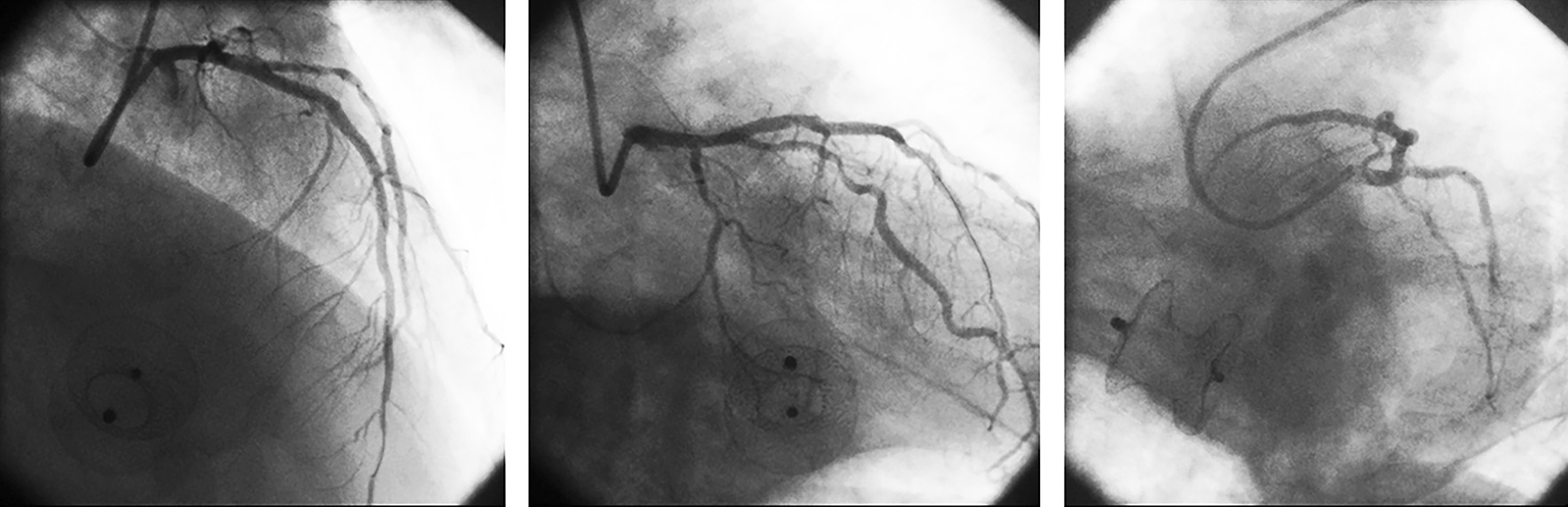
In secondary PCI, 2 stents (TiVoli^TM^ 2.5 × 35 mm and TiVoli^TM^ 2.75 × 30 mm) were placed for LAD, and 1 stent (Partner^TM^ 2.5 × 36 mm) for LAD-D_1_ with culotte technique. The three panels are cranial, caudal, and spider views, respectively

Two days later, the augmentation ratio was decreased to 1:2 and then to 1:3. IABP was weaned off on day 31. Secondary PCI was conducted on day 35 for LAD lesions (Fig. 5). The patient was discharged on day 41.

At the last follow-up 6 years later, CAG and TTE revealed no in-stent restenosis, no left-to-right shunt, no mitral regurgitation, and 51% LVEF. He kept taking aspirin, atorvastatin, and metoprolol regularly as the medication therapy.

All procedures performed in this study involving human participants were in accordance with the ethical standards of the institutional and/or national research committee(s) and with the Helsinki Declaration (as revised in 2013). Written informed consent was obtained from the patient.

## Discussion and conclusions

In this case, the VSR caused by AMI was successfully managed with deferred percutaneous closure. The 28-day waiting period was supported by IABP, and allowed the infarcted myocardium around the perforation to mature for eventual closure. The critical role of prolonged IABP was reflected by rapid hemodynamic stabilization after the rupture and the failure to wean off from IABP on day 12.

The pathologic hallmark of VSR is necrosis of the ischemic myocardial tissue, followed by neutrophilic infiltration, thinning and eventual rupture of the septum [3]. The occurrence of VSR complicating anterior myocardial infarction accounts for about 60%, and the occurrence complicating inferior myocardial infarction accounts for 40% [20]. Immediately after VSR, tissues surrounding the rupture site are retracted, friable, and defective [21]. Prognosis tends to be poor after early repair, and improves significantly if the intervention is deferred by 2 weeks or longer due to the maturation of the VSR, recovery of myocardial function, establishment of collateral blood supply, and adaption to hemodynamic changes [7, 12, 18]. A variety of measures of immediate cardiac support have been used to stabilize hemodynamics prior to the deferred operative intervention [13].

IABP is an important tool for hemodynamic stabilization in high-risk patients [10, 22]. In patients with VSR, IABP could also increase coronary perfusion to allow ischemic area around the VSR to heal [9]. In this case, the patient received prolonged IABP support prior to transcatheter VSR closure. Immediate repair is not recommended after AMI since myocardium is too fragile. A waiting period of 3–6 weeks before surgery allows the margins of the infarcted myocardium to develop a firm scar to facilitate surgical repair [19]. The presence of left-to-right shunt, however, compromises hemodynamic stability. IABP and other mechanical circulatory support during this waiting period have been reported in several studies [9, 23].

In the prethrombolytic era, outcomes after septal rupture were extremely poor, with 90% in-hospital mortality in medically treated patients [24]. With immediate or delayed perfusion operation, in-hospital mortality in such patients now stands at approximately 50% [25]. In AMI patients, VSR typically occurs in two windows: the first 24 h and then 3–5 days [3]. In comparison to repairing congenital ventricular defect, transcatheter closure of VSR in AMI patients, who are poorly tolerated in the settings of AMI and sudden-onset cardiac shunt, is more challenging. We encountered malignant arrhythmias but were able to proceed with the help of a temporary pacemaker and continuing IABP support. Besides, the morphology of VSR is usually irregular, discrete, and changeable throughout the cardiac cycle, while a ventricular defect is nearly round or oval-shaped.

The patient had already completed reperfusion therapy and was under DAPT, thus transcatheter closure is an effective option in the timing of stable hemodynamics [26, 27], but the DAPT is not a contraindication of surgical repair. Surgical repair was once considered but abandoned because of a higher risk of general anesthesia, extracorporeal circulation support, and bleeding when DAPT was adopted. Absence of scientific head-to-head comparison, what is the first choice for VSR patients, surgical repair or percutaneous closure, remains a question for all cardiological professionals [26]. Here are the pros and cons we summarize about surgical and percutaneous operation of VSR. The pros of surgical repair are directive operation recommended by guidelines, more reliable closure of the perforation, zero X-ray exposure for both patients and medical staffs, and sophisticated technique with a longer history. The cons of the surgery are massive invasion, general anesthesia required, and extracorporeal circulation support needed. The pros of percutaneous closure are minimal invasion and only local anesthesia needed. The cons of percutaneous closure are fluorescent exposure, shorter history of implementation, and occurrence of residual shunt, hemolysis, and device dislocation and/or embolism due to the irregular shape of VSR, which are the pitfalls and risks of previous transcatheter closures reported [7, 26, 28]. For this patient, the myocardium was still fragile, and a conventional guidewire and catheter operation through the rupture can induce malignant arrhythmia endangering the patient’s life. There are some risks including: occluder dislocation due to the friable surrounding myocardium, vascular injuries by catheter, valve dysfunction affected by the occluder, myocardial injuries causing pericardial tamponade, and an occurrence of complete left bundle branch block after the occluder implantation. We had a similar experience of a postinfarct VSR patient who was assisted with IABP for 21 days, and then received surgical repair of VSR and coronary artery bypass graft (CABG) successfully. The patient remained hemodynamically stable in the perioperative period with the support of IABP. So, the optimized decision for postinfarct VSR patients ought to be led by the multidisciplinary heart team.

In conclusion, deferred closure of VSR may have a better outcome for patients who respond well to aggressive therapy. Prolonged implementation of IABP can be a viable option to allow eventual closure of VSR in AMI patients. Transcatheter closure may be considered as a second choice for the selected senior and vulnerable patients, but the risk is still high.

## Data Availability

The datasets supporting the conclusions of this article are available in the GitHub repository, https://github.com/YangChuan80/CaseData_VSR.

## Abbreviations

VSR: Ventricular septal rupture
AMI: Acute myocardial infarction
PCI: Percutaneous coronary intervention
IABP: Intra-aortic balloon pump
HR: Heart rate
BP: Blood pressure
DAPT: Dual anti-platelet therapy
CAG: Coronary angiography
RCA: Right coronary artery
LAD: Left anterior descending artery
LCX: Left circumflex artery
CCU: Cardiac intensive care unit
rhBNP: Recombinant human brain natriuretic peptide
TTE: Transthoracic echocardiography
NYHA: New York Heart Association
LVEF: Left ventricular ejection fraction
CABG: Coronary artery bypass graft
